# On the Diagnosis of Pulmo-Tuberculosis

**Published:** 1849-03

**Authors:** Austin Flint

**Affiliations:** Prof. of Principles and Practice of Medicine, and Clinical Medicine; Med. Dep't. Univ. of Buffalo


᠎# BUFFALO MEDICAL JOURNAL

AND

### MONTHLY REVIEW.

VOL. 4. MARCH, 1849. NO. 10.

### ORIGINAL COMMUNICATIONS.

ART. I.—*On the Diagnosis of Pulmo-Tuberculosis. A Clinical Lecture.* By AUSTIN FLINT, M. D., Prof. of Principles and Practice of Medicine, and Clinical Medicine; Med. Dep't. Univ. of Buffalo.

GENTLEMEN:—During the past three months of clinical instruction, at this Institution, your attention has been directed to numerous cases of tuberculous disease of the lungs, at the hospital and dispensary, and I have endeavored to avail myself of the opportunities which these cases have afforded, to illustrate the positive and relative significancy of the phenomena involved in the diagnosis of this disease. In view of the scientific interest and practical importance of the subject, I purpose, on this occasion, to recapitulate the more important of these phenomena, and to consider, briefly, their character, and the specific value which they possess, separately and combined, as elements of diagnosis. We shall thus not only review, but associate considerations which have heretofore been presented disconnectedly as suggested by the various features of different cases. Such a retrospection will also prepare us to study more advantageously other cases of the disease which will come under notice during the remainder of the session.

For all purposes of practical instruction in diagnosis, pulmo-tuberculosis may be divided into two stages. The first stage will embrace that period (differing widely in different cases as regards time) during which the tuberculous deposit remains in a solid, or crude state—the stage of *crude tuberculization*, as it has been called. The second stage (also quite indefinite in duration) will embrace the period of the disease which elapses after softening, ulceration, and expectoration of the tuberculous product, have taken place. The diagnosis, in either stage, is based upon phenomena determined by rational and physical methods of investigation. It is, however, with reference to physical signs, more especially, that this division into two stages is convenient. I will, in the first place, direct your attention to the physical signs belonging to each of the stages in succession, and, afterward, to the vital phenomena appertaining to both stages, the latter phenomena being usually distinguished as *rational symptoms.*

*Signs Derivable from Percussion in the First Stage.*—*Pulmo-tuberculosis is characterised by the deposition in the pulmonary tissues of the heterologous morbid product called tubercle. In proportion as this solid material accumulates, the lungs undergo an important physical change. The normal vesicular structure is compromised, the collective capacity of the cells to contain atmospheric air being diminished. This is sufficiently obvious. Now, how is the existence, and the degree of this physical change to be ascertained and measured by percussion? It is evident, that if the relative proportion of the aeriform and solid contents of the thoracic cavity be disturbed in any considerable degree, the sound yielded by percussion should indicate such a change. There must be more or less diminution of the resonance proper to the normal condition. A duller resonance over any part of the chest than belongs to that part in its normal state, is physical evidence that the proportion of solid matter over the gaseous contents, has become predominant. The degree of the dullness, other things being equal, indicates the degree to which the solid material has encroached upon the vesicles containing air. The problem then remaining is, to determine whether this solid material be tubercle; but the question may first arise, is it easy, in all cases, to recognise the presence of abnormal dullness, and to estimate its degree? There is not, it is true, a fixed intensity of resonance belonging to every healthy chest. Different persons differ in this respect, owing to various circumstances. At first view, it would seem that this fact would materially impair the value of percussion. It does not, however, because we never measure resonance by reference to an ideal standard, but by comparison of the two sides of the chest in the same individual. It is a law of pulmo-tuberculosis that it attacks one side of the

---

* The term *pulmo-tuberculosis* is submitted as expressing the character of the disease better than *phthisis*, the etymological signification of the latter being objectionable, not less than the popular name *consumption.* Pulmo-tuberculosis denotes, what the disease is, tuberculous disease of the lungs.

chest first, always advancing more rapidly in one side than in the other. Hence, the practical point in diagnosis is, not to ascertain whether the sound emitted by percussion is less than it is in the average of individuals, but whether over corresponding regions of the two sides of the chest, it is equal, or unequal. Fortunately for the availability of percussion in diagnosis, the normal resonance of corresponding regions of the two sides of the chest is similar in the same individual. The exceptions to this rule are very rare, and generally explicable by obvious circumstances. Recollect, then, that you have not to determine whether the resonance on percussion is in an *absolute* sense, clear or dull, but whether it be *relatively* so.

But other physical causes than the presence of tuberculous deposit may occasion dullness of resonance, and how are we led to attribute it to the existence of the latter as the cause? Here we are to take into view another general law of the disease; and, also, general laws of other diseases occasioning diminished resonance. Other pulmonary affections attended with diminution, or absence of resonance, are Pneumonitis, and Pleuritis with effusion. Now, the tuberculous deposit almost invariably commences at the apex of the lung, and accumulates especially in the upper lobe. Hence, the physical signs determinable by percussion, are chiefly manifested at the summit of the chest. On the other hand, Pneumonitis as invariably, in the adult, attacks the lower lobe, or lobes, producing flatness over the inferior, or middle third of the chest. The effusion attending Pleuritis gravitates to the lowest portion of the pleural cavity, and is first apparent at the inferior portion of the chest, the flatness extending upward, in proportion as the quantity of fluid accumulates. Another point of distinction is, that the diminished resonance from Pneumonitis, and the effusion of Pleuritis, is more complete. The sound is not *dull*, but *flat*, while the tuberculous deposit seldom takes place so abundantly as to solidify the lung, to the same extent, and, consequently, the resonance is rarely entirely extinct, but only more or less diminished.

In view of the foregoing laws, dullness on percussion over the upper portion of the chest, on one side, considered relatively to the other side, furnishes strong presumptive evidence of the presence of the tuberculous formation. As respects degree of dullness, it may have, in a series of cases, every shade of intensity, from an amount scarcely appreciable, to an approximation toward actual flatness, the variations corresponding, of course, to the degree and extent of consolidation. When the latter are considerable, the disparity of resonance in the two sides is at once apparent. The first tap of the finger suffices to reveal the fact. But when the tuberculous formation is in small isolated masses, or is limited to a small portion of the pulmonary structure, or situated centrally in the lung, it is by no means so easy to decide on the existence of diminished resonance; and, to derive information from percussion, under these circumstances, requires tact, and great care. Much will often depend, as respects diagnosis, upon the decision of the question whether dullness exists, or not. The clavicular, infra-clavicular, and inter-scapular regions, are to be repeatedly explored, for it is in these regions that a disparity in resonance is to be expected. We should also carefully compare the resonance developed by percussion on the top of the shoulder, just above the post-clavicular or acromial space, and over the scapula above the spinous ridge, on both sides. You will recollect cases, among those which have been presented during the session, in which distinct dullness was apparent at the latter points, when, in the regions previously mentioned, scarcely a difference in the two sides was appreciable. I would impress the importance of recollecting this practical point.

You will perhaps inquire, how early in the progress of tuberculosis may the resonance of the chest be modified so as to be distinctly appreciable by percussion? While tubercles are small, disseminated, and separated by healthy vesicles, the sound may continue to appear perfectly normal. The change is, as yet, too slight to be detected by the ear. It may happen, also, that the two lungs on both sides become affected in quick succession, and that, in the progress of the disease, one side is but little in advance of the other. Hence, the difference in resonance may not be obvious. But in a large proportion of the cases of this disease which come under our notice, percussion, at once, affords evidence sufficiently clear and explicit, and we are able often to estimate with considerable precision the amount of solidification, by the contrast in resonance which the two sides of the chest afford.

*Signs Declared by Inspection.*—Inspection may furnish valuable signs even in the first stage. The deposit of the tuberculous product in considerable quantity, frequently occasions condensation or contraction, the portion of lung tuberculized, occupying less space than the distended normal vesicles of the same portion. Hence, occurs depression in the post-clavicular, or infra-clavicular spaces, and, sometimes, flattening of the upper third of the chest. It is needless to say, that the diagnostic value of this sign chiefly arises from its being present on one side of the chest, as compared with the other side.

Diminished mobility of one side is another sign. On placing the patient in a good light, and attentively contemplating the chest, the upper portion of one side may be found to undergo less expansion during inspiration, than the other. This shows that the internal distending agent, the atmosphere within the vesicles, has suffered diminution. It may, also, in some cases in part, be due to adhesions over the tuberculous deposit.

Among the cases which have come under our notice, as you will recollect, several have exhibited both these signs, and they are usually associated together, in the same case.

*Signs derived from Auscultation.*—One of the excellencies of physical signs consists in the fact, that they mutually confirm, or correct each other. In the diagnosis of pulmo-tuberculosis, the evidence of percussion, although highly valuable, is often imperfect and inadequate, and if exclusively depended on, might sometimes lead to erroneous conclusions. This can seldom occur, if, in connection, proper attention be devoted to exploration by auscultation. Moreover, the auscultatory signs are sometimes sufficient of themselves, in so far as physical diagnosis is concerned, in cases in which percussion furnishes little information, or none whatever.

The auscultatory signs involved in the diagnosis of the early stage of tuberculous disease, are several in number. Some of them are by no means striking in themselves, but acquire significance and value from incidental circumstances. Considered separately they would appear to claim little notice, but taken in connection with other facts, they possess hardly less importance, than if they alone furnished specific indications. The first of which I will speak, is *tubular respiration*, or, as it is sometimes termed, *bronchial respiration.*

Whenever the pulmonary structure is solidified, by deposit of tuberculous matter, or fibrin, or by compression, the soft, expansive, breezy murmur produced by the dilation of the pulmonary cells in consequence of the entrance of the air in inspiration, ceases to be heard over the region of the chest corresponding to the solidification. The larger bronchial tubes, however, remain unobstructed: air circulates through them with freedom. What is the result upon auscultation? The vesicular murmur is nearly or quite extinct, and the sound produced by the currents of air in the bronchial tubes, takes its place. In a normal condition of the lung, the latter, or bronchial sound, is not heard, because, first, the vesicular murmur overpowers it, and, second, because the air cells, filled with air, which are interposed between the bronchial tubes and the ear of the auscultator, form a poor conducting medium for the transmission of sound. But these air cells being converted into a solid by tuberculous, or other deposit, the bronchial sound is now not supplanted by the vesicular murmur, and is conducted with more facility to the ear. This is the mechanism of the bronchial or tubular respiration, which is generally heard over considerable deposits of tuberculous matter.

The tubular respiration is heard with more intensity over the larger bronchia. In parts somewhat remote from large bronchial tubes, for example in the infra-clavicular region close to the clavicle, and near its acromial extremity, the auscultatory sign of consolidation may be simply the absence of all respiratory murmur.

Beginners in auscultation are apt to fancy that the tubular respiration is necessarily louder than the vesicular murmur; but this is not the case. The distinction concerns the character, not the intensity of the sound.

It is heard generally both in the acts of expiration and inspiration, or rather, the sound of expiration is longer, compared with that of inspiration, than when the normal vesicular murmur is present. This fact will be of assistance in the discrimination, until the ear becomes habituated to the intrinsic difference. Instead of being in the ratio of one to three, or five, the expiratory tubular sound may be prolonged to nearly, or quite the length of inspiration.

Next, of *rude* or *rough respiration.* Suppose tuberculous formation to have taken place, not in a sufficient degree, or to a sufficient extent, to occasion well defined *tubular* respiration, but sufficiently to occasion an appreciable modification of the vesicular murmur. There is then an approximation to the tubular character of sound, and this approximation constitutes what is called *rudeness* or *roughness.* In connection with this modification, the expiration may be somewhat prolonged, which will here, as in the former instance, assist us in the discrimination. This rudeness, or roughness, may, in some cases, be appreciable before an accumulation of tuberculous matter takes place sufficient to occasion dullness on percussion. Hence, it is to be regarded as a valuable diagnostic sign of incipient tuberculosis.

In connection with both *tubular* and *rude* respiration, the stethoscopic character of the voice should be ascertained. Bronchophony is by no means a constant attendant upon the former, although due to the same physical condition, but it frequently co-exists, and, when present, will go to confirm the fact of tubular respiration. So, an increased vibration of the voice through the cylinder may be associated with *rudeness,* but not amounting to well defined bronchophony. A vibratory thrill perceptible to the touch (vocal fremitus) also occasionally co-exists, and, therefore, is to be sought for under the same circumstances.

Several other signs belong to the early stage of pulmo-tuberculosis, which

I will mention, briefly explaining the reason of their significance, One of these is a *prolonged expiratory murmur*, even when the character of the respiration may neither be tubular, nor rude. The attention of the profession was first directed to this sign by the late lamented Dr. James Jackson, Jr., of Boston, Mass. Its value is now admitted by the best auscultators. The most rational explanation would seem to be, that the presence of small tubercles, by pressure upon the capillary bronchial ramifications, retard the egress of air from the vesicles, thus prolonging, and possibly, at the same time, developing an increase of sound. Another sign is, *absence of the vesicular murmur, and of all sound.* Another is, a *sibilant rale* and occasionally a *click* at the upper part of the chest. Another is, a slight *sub-crepitant rale* at the end of the inspiratory act, at the upper part of the chest. So far as my experience goes, the latter is a frequent sign of incipient tuberculosis. The value of each of the three last mentioned signs is dependent wholly on incidental considerations, not on their intrinsic character. They are all probably due to sub-acute bronchitis, or, possibly, merely bronchial irritation, situated in the capillary bronchi, in the immediate vicinity of the tuberculous deposit, and occasioned by the proximity of this deposit. Why do phenomena indicating thus capillary bronchitis, or bronchial irritation, become evidences of tuberculization? Because both capillary bronchitis and circumscribed bronchitis, as original affections, very rarely occur. Probabilities are vastly against the supposition of their occurrence except as consequences of the deposit of tuberculous matter. Hence, they may become signs of the presence of tuberculous formation, even before this deposit is sufficient to occasion other positive signs. *But to be signs of tuberculosis they must be limited to a circumscribed space, and that space, in the vast majority of cases, the infra-clavicular region.* Their significance, also, is enhanced, if they are presented on one side of the chest only, or, at least, if they are more marked on one side than on the other. Observe, that these signs are not only intrinsically different, but some of them even opposite in character; but it is not the character of the sign which gives it diagnostic importance, but its being restricted to the summit of the chest, on one side, and confined to a circumscribed space. Persistency, should also be included among the conditions of significance. Hence, we are to satisfy ourselves of the continuance of one or more of these signs by repeated explorations.

Another sign remains to be mentioned. It is, an abnormal audibleness of the sounds of the heart at the summit of the chest, on the left, and even on the right side. The explanation of this is, that solidified lung being a better conductor of sound than the pulmonary vesicles filled with air, the sounds of the heart may be heard over a tuberculous deposit in regions where, if the lung were normal, they could not be heard at all, or with much less distinctness. This is by no means available as a constant, or prominent sign, being dependent on continuity of the tuberculous deposit so as to be in proximity to the heart. And when it is present, it co-exists with other signs more definite and reliable. But, as affording confirmatory evidence of consolidation, it is well to bear it in mind. I have directed attention to this, among other signs, in some of the cases which have been examined during the session.

Let us now briefly review the several signs belonging to the first stage of pulmo-tuberculosis, with reference to their mutual relations, and to their bearings, separately and combined, upon diagnosis. In a large proportion of the cases of this disease which present themselves, we are able to discover more or less relative dullness on percussion at the summit of the chest, in the clavicular, or infra-clavicular regions. In a certain proportion of the cases in which distinct dullness is not distinctly demonstrable in the regions just named, we shall be able to discover it *above* the clavicle, or upon the scapula. In many cases, to make out this sign requires very little expertness of manipulation, or delicacy of discrimination. In other cases, where the disparity in the two sides of the chest is slight, experience, tact, and a cultivated ear are important. A good auscultator will be able, in some instances, to determine on the presence of this sign, when one less practiced, or less apt, would fail. The presence of relative dullness at the summit of the chest is strong presumptive evidence of tuberculosis; we are not, however, to base our diagnosis on this sign alone, by any means, but to obtain farther evidence by other physical signs, as well as by rational symptoms. The absence of dullness is not conclusive evidence against the existence of tuberculosis. Tuberculous deposit may be present without producing an appreciable modification of resonance, but determinable by other signs, and by symptoms.

In connection with dullness on percussion, we may expect to find, on auscultation, *tubular*, or *bronchial* respiration; or, an approximation thereto, the latter giving rise to *rude* or *rough* respiration, these signs being sometimes accompanied with bronchophony, or an approximation thereto. In portions of the chest remote from large bronchia, even when dullness is discernible, there may be simply extinction of the normal respiratory murmur. The degree of tubular character which the respiration assumes, will depend upon the situation of the consolidation, as respects the larger bronchia, and its superficial location as repects the lung. The presence of either of these auscultatory signs, in conjunction with dullness on percussion, renders the evidence for tuberculization greater, and, indeed, in most instances, very conclusive.

In combination with these signs, also, we have, in a certain proportion of cases, confirmatory evidence disclosed by inspection, viz., *depression of the infra-clavicular, or post-clavicular spaces,* and, sometimes, *flattening of the upper third of the chest.* All these signs, it will be borne in mind, depend for all the diagnostic significance which they possess, upon the fact of their being present on one side of the chest as contrasted with the other side, or, at least, more strongly marked on one side.

The other auscultatory signs that have been enumerated are, a *sibilant rale; marked feebleness, or absence of the respiratory murmur; prolonged expiration; a soft sub-crepitant rale, and exaggerated audibleness of the sounds of the heart.* One or more of these signs may accompany those previously mentioned. Indeed, they may all be presented in the same case, either simultaneously, or successively. The diagnostic importance of the latter, not less than the former signs, depends on their being limited to the upper portion of the chest, generally quite circumscribed; confined exclusively to, or more strongly marked on one side, as contrasted with the other side; and, singly or successively, persistent in the same place, not observed on a single examination only, but demonstrated by repeated explorations. Associated with dullness on percussion, and tubular or rude respiration, the signs last mentioned will serve to confirm a conclusion already pretty certainly established. But they may posses importance beyond this. In some cases in which tuberculization is not demonstrable by percussion, and by the development of tubular, or well marked rudeness of respiration, these latter signs, singly or collectively, may, when taken in connection with rational symptoms, render the presumption of tuberculosis very strong, if, indeed, they may not suffice for a positive diagnosis. In this point of view, they possess great importance, affording valuable aid at that period of the disease when it is of most practical importance that it be discriminated—viz., its incipient stage.

I pass now to *signs belonging to the second stage of pulmo-tuberculosis.*

It is to be remarked, that the signs belonging to the first stage, may also be continued into the second stage, in a greater or less degree; for all the tuberculous product which a lung contains, does not pass through the processes of softening and evacuation simultaneously. These processes advance in some portions more rapidly than in others. Moreover, the tuberculous deposit takes place gradually, or in successive epochs, and fresh deposits may be going on even after cavities have formed. A lung affected with tuberculization, exhibits, generally, after death, the disease in every stage of its progress. Hence, the signs of the first stage, are more or less intermingled with those of the second. . We are to mention, as the signs belonging to the second stage, only those that are peculiar to it. And these will claim but a cursory notice, for they are intrinsically simple, easily appreciated, and unequivocal in their character. The diagnosis at this stage is comparatively easy.

The signs characteristic of the second stage declared by percussion are, *an improvement in resonance compared with the preceding stage*, owing to the evacuation of solid tuberculous material, leaving cavities filled with air, (consequently becoming more resonant) and to greater thinness of the parietes of the chest, from emaciation. The sound on percussion will vary at different times, in proportion as the cavities are more or less filled with morbid secretions. Occasionally, over a circumscribed space, percussion developes a very clear and tympanitic resonance, denoting the existence of a large cavity superficially situated. And, under these circumstances, the sound is sometimes found to have a musical intonation, called, by the French, the *bruit de pot fele*, which, in English, is rather inelegantly rendered, *the sound of the cracked pot.* I have met with a case in which *inspection* clearly indicated the existence of a large superficial cavity, the intercostal spaces being visibly distended over a circumscribed space during acts of coughing, and a sense of fluctuation, at the same time, communicated to the touch.

On *auscultation*, in this stage, we have, when the cavities are empty, or nearly so, the *cavernous respiration*, which in its intrinsic character is identical with the *tubular respiration*, but more intense than the latter, and more circumscribed. The degree of intensity will depend on the size of the cavity, or cavities, the contiguity to the superficies of the lung, and the freedom of communication with large bronchia. Occasionally the cavernous sound presents a musical intonation, resembling the sound produced by blowing lightly into the mouth of a vial, and probably owing to the same mechanism. This variety of the cavernous respiration has received the name of *amphoric*.

Accompanying the cavernous sound in respiration, is that sensation, when the patient speaks, as if the voice entered directly from the mouth through the stethescope to the ear of the auscultator; sometimes heard with a vividness that is almost painful. We have, then, the vocal sign called *pecto-*

*riloquy*, the pulmonary sign first noticed by Laennec, leading the way to the discovery of auscultation. This sign, however, it is to be remarked, is not constantly observed even when cavernous respiration is present.

These signs imply that cavities are nearly, or quite empty. If more or less filled with the secretions which constantly take place from their lining membrane, and which accumulate, especially at night, we have, *coarsely crepitating rales*, if the cavities are small, and, if the cavities are large, *gargouillement*, or *gurgling*, occasioned by the motion of the liquid contents of the cavities by the current of air received by inspiration, and by explosive bubbles of air, somewhat as in ebullition.

In connection with these signs we have more or less *mucus* or *bronchial rales* from the presence of secretions in the bronchial tubes, the latter, however, possessing no peculiar significancy.

With this brief notice of the physical signs proper to the second stage, I pass to the third division of the subject, which embraces other events occurring in the progress of the disease under consideration, than those belonging to physical exploration—in other words, the *rational symptoms involved in the diagnosis of Pulmo-tuberculosis.*

In this branch of the subject my object is by no means to undertake a descriptive history of the disease, but simply to recal to your minds some of those points which we have found to be specially significant as bearing on the diagnosis. Even in this aspect, due regard for brevity will not permit me to treat of the subject so fully as its merits might claim. I will simply enumerate the symptoms which possess most importance, with short annotations upon each. Recollect that we are now to speak of the diagnostic value of rational symptoms, considered without reference to physical signs. And, *first*, of *cough*. The most important diagnostic consideration as respects cough, relates to its origin, and early history. The question of diagnosis will generally embrace tuberculosis, and sub-acute bronchitis. Now, sub-acute bronchitis usually follows acute bronchitis, which will, of course, have been attended by painful cough, followed by expectoration after two or three days, etc. But, in a large proportion of cases of tuberculosis, the cough, when first noticed, was short, and dry, so continuing for a greater or less period, gradually increasing, as also the expectoration, the latter being often insignificant in amount for a considerable time. In short, the origin and early history of a large proportion of cases of tuberculosis, do not present the evidences of acute, or even sub-acute bronchitis. Consequently, if we find cough to have been developed without the evidences of sufficient bronchial inflammation to constitute an attack of bronchitis, this is entitled to have considerable weight among the assemblage of rational symptoms pointing to tuberculization. We are always to consider, in this connection, that cough is occasionally due purely to nervous disorder—but it will then be found associated with other evidences of neuropathia, or hysteria, and usually with spinal irritation, the latter, so far as my experience goes, being seldom conjoined with a tuberculous diathesis.

It is to be remarked, that although the commencement of cough without evidences of bronchitis be a symptom of tuberculosis, the fact of its being accompanied by the evidences of bronchitis is, by no means, to influence our decision that it is *not* a symptom of tuberculosis, inasmuch as, in a certain proportion of cases, the tuberculous deposit does accompany, or follow well marked bronchial inflammation.

There are other circumstances appertaining to cough which would claim attention in a full consideration of the subject, but they are secondary in importance to the point just noticed.

*Second*, of *Expectoration.*—There are several circumstances connected with the expectoration which are to be considered as favoring the opinion that tuberculous deposit has taken place. I will mention those which seem to me as most worthy of notice:—

*a.* A scanty, transparent, frothy expectoration for some time after the commencement of cough. The significancy of this depends on the evidence which it affords that the cough and expectoration were due to irritation occasioned by the presence of tubercles, not as the effects of bronchitis irrespective of tuberculosis. Cases not unfrequently present themselves in which tuberculization has taken place sufficient to produce well marked dullness on percussion, the cough remaining slight, the expectoration small in amount, and of the character just stated.

*b.* The occurrence of sudden increase of expectoration, its appearance, at the same time, puruloid; this occurrence taking place once, or successively. This denotes the evacuation of a collection of softened tuberculous matter. The fact of pus corpuscles being contained in the expectoration is now shown to have, in itself, little importance as a diagnostic symptom, since they may be furnished by the bronchial mucus membrane, as well as by the cyst lining pulmonary cavities. But if pus be expectorated under circumstances which render the existence of bronchitis improbable, it becomes a valuable symptom.

*c.* The expectoration of tuberculous matter imperfectly liquified. This is an event extremely rare, so that pathologists of very large opportunities for observation, have never met with it. It has occurred, however, in an instance at the hospital during the present course. The patient presented all the evidences, physical and rational, of tuberculous disease, the expectoration being, habitually, tolerably copious, and puruloid. Suddenly, after a violent effort of coughing, he expectorated several sputa which arrested his attention, being unlike anything that he had heretofore expelled, and it was accordingly preserved for my inspection. It consisted, apparently, of tuberculous matter, being in small lumps, the largest of which was about the size of a pea, not round but angular, very friable, like old cheese in this respect; of an ashy white color. The quantity of this expectoration examined, which was commingled with some mucus, would have filled a teaspoon. A similar expectoration never afterward occurred in this case, and he continued under observation several weeks. Simultaneously with the evacuation of this matter, the physical evidences of a cavity were found in the left infra-clavicular region, which had not previously attracted notice.

The occurrence of tuberculous matter, with the physical characters distinctive of it, is, however, too rare to be depended on as a symptom of the disease. Prof. Chomel states, that during thirty years of close attention to the sputa of patients with tuberculosis, he has not been able to satisfy himself of the presence of tuberculous fragments in a single instance.

*d.* Copious expectoration of purulent sputa at once, or in a short time, and, afterward, comparatively little expectoration for several hours. This is a prettty good symptom of the existence of a cavity, or of cavities, of considerable size, admitting of the accumulation of secretions until efforts are provoked for their expulsion, after which, some hours elapse before a similar accumulation again takes place. This copious expectoration generally occurs in the morning, accumulating during sleep, and frequently a collection to such extent does not occur during the daytime, the cavities being kept empty by expectoration of their contents nearly as fast as their secretion takes place.

Other points relating to the expectoration might be mentioned, but I pass to speak—

*Thirdly*, of *pain*.—In the autopsies of subjects dead with pulmo-tuberculosis, adhesions of the pleura costalis and pleura pulmonalis are almost invariably found. They extend more or less over the whole surface of the membrane, are more or less firm, generally, in the latter respect, differing in different portions. They are evidently of an old date, or, at all events, not recent, and, in fact, have taken place at successive periods of the disease, over different collections of the tuberculous deposit. These adhesions, occurring as just stated, are evidences of a series of attacks of sub-acute, dry, circumscribed pleuritis, which are often indicated at the time of their occurrence by lancinating pains of greater or less severity, and sometimes by febrile movement. In some cases these pleuritic attacks are accompanied by so slight a degree of pain as hardly to attract attention, in other cases they give rise to symptoms which are prominent. They so uniformly enter into the history of the disease, and are so generally denoted by more or less pain at successive periods, that the occurrence of *pleuritic stitches* becomes an important diagnostic feature of pulmo-tuberculosis. In taking the history of cases, you will have observed that I am careful to obtain definite information whether the patient has repeatedly experienced these pains, and in commenting on the rational symptoms with reference to the diagnosis, I have frequently called your attention to this point. The pains are referred to different regions of the chest, at different times, frequently beneath the scapula, or in the shoulder. At the time of their occurrence they are often accompanied by tenderness upon pressure. Percussion in some persons is painful, owing to this cause.

*Fourthly, Chills.*—In a large proportion of cases, distinct chills, or marked chilliness at times, characterize the rise and progress of the affection under consideration. The occurrence of this symptom, therefore, forms an item of evidence in the diagnosis, and inquiries with respect to it should not be omitted. In some instances, in the incipiency of the disease, chills occur, and recur daily, for sometime, with all the regularity of an intermittent. The febrile movement which succeeds is not prominent, and perspiration may not follow. Under these circumstances, while the pulmonary symptoms are so slight as not to have attracted much notice, the case may be regarded as one of intermittent. I have committed this mistake, and know of its being committed by others. Quinia, I may remark, under these circumstances, is not an improper prescription, and will arrest the paroxysms.

*Fifthly, Hæmoptysis.*—The observations of Louis, and others, have shown this symptom to enter so frequently into the early history of tuberculosis, and to occur so seldom independently of this disease, that it possesses important diagnostic value. It may occur wholly irrespective of tuberculization, as my own observation have abundantly satisfied me; but, yet, even if it were the first and, at the time, the only symptom, I should regard it as furnishing strong grounds for suspicion. Associated with other pulmonary symptoms it is entitled to great weight. It is explained by the local congestion produced by, and perhaps existing prior to, the deposit of the tuberculous product. It is important, of course, to ascertain that the hemorrhage is from the lungs, not from the stomach, pharynx, or posterior nares. We are also to satisfy ourselves that it is true hemorrhage, not merely mucus secretions streaked, or tinged with blood.

*Sixthly, Accelerated or Embarrassed Respiration.*—If the tuberculous deposit have taken place in considerable quantity, and, especially, if it have taken place rapidly, the respiration will usually be affected. The respiratory acts will be more or less increased in frequency; there will be some habitual labor of breathing, together with more panting on exertion, as in walking, ascending stairs, etc., than is explicable by reference to the circulation, or muscular debility. The *alæ nasi* frequently indicate embarrassment of respiration. Generally, the patient does not complain of dyspnœa, so that we must determine the abnormal deviations from health, in this particular, by our own observations. The respirations should be enumerated, and carefully examined, both when the patient is tranquil, and directly after some muscular effort.

*Seventhly, Emaciation.*—The functions of assimilation deteriorate from the commencement of tuberculous disease, as evinced by impaired nutrition. Emaciation may go on more or less rapidly, but it is progressive. What is lost, is not regained. The exceptions to this law are so few, as hardly to militate against its correctness for all practical purposes of diagnosis. Constant, although very gradual diminution of weight, therefore, is evidence in favor of this disease, and definite information should be obtained, if practicable, with respect to this point.

*Eighthly, Pallor of Complexion.*—Marked pallor of the complexion is so common a concomitant of tuberculosis, as to deserve being included among the evidences of this disease.

*Ninthly, Accelerated Circulation.*—An abnormal frequency of the heart's contraction is a very constant symptom of tuberculosis, although, as respects degree, there is much variation in different cases. The pulse is usually more or less accelerated, and this is a persistent symptom. The frequency even may amount to, or exceed 100, before many of the unequivocal evidences of the disease are developed. The pulse also exhibits a quickness, and vibratory character, in many cases, which is peculiar, giving rise to what has been aptly designated "tuberculous fever." Permanent acceleration of pulse, especially with the peculiarities just referred to, is one of the most reliable of the rational evidences of this disease. I have also been led to regard it as possessing more importance than any other single symptom, as a criterion of the rapidity of progress, which the disease is making. I think we can form an opinion with regard to the probable duration of the disease, better from this symptom, than from any other. If the pulse be notably and persistently frequent, we may be assured the disease is progressing rapidly, and will run comparatively a short career.

*Tenthly, Diarrhœa.*—I do not now refer to the colliqative dirarrhœa incident to the last stage of this disease. In its earlier periods there is a tendency to diarrhœa in many cases. It occurs at intervals, and is usually of short duration, subsiding spontaneously, or relieved by remedies. This is sufficiently common to entitle the fact of repeated attacks of diarrhœa to be included in the present category of diagnostic symptoms characterising this disease.

*Eleventh. Hereditary Predisposition.*—This can hardly be included, with strict propriety, among the *symptoms* of tuberculosis, but a family tendency to the disease should not be overlooked in the diagnosis. That such a tendency may be inherent in the constitution, and be transmitted to offspring, are facts abundantly established. It has also been observed to pass by one generation, and reappear in the next. The point, therefore, should be ascertained, in all cases, whether progenitors, immediate or remote, have been affected with tuberculous disease, and if there are grounds to suspect the existence of an inherited predisposition, this fact should add weight to the probabilities derived from symptoms and signs. In the diagnosis of the incipient stage of the disease this point possesses considerable importance.

---

I have thus, gentlemen, passed in review the more important of the phenomena, rational and physical, involved in the diagnosis of pulmo-tuberculosis. I have omitted some of the more obvious and unequivocal evidences which are presented in the last stage, or after the character of the affection is sufficiently declared, for example *hectic paroxysms, œdema of the extremeties, dyspnœa,* etc., my object having been to direct your attention to those elements which it may be important to take into account while the diagnosis is a matter of question. Of the importance of a correct diagnosis it would be superfluous to speak. It is to be borne in mind that the discrimination is important in a two-fold aspect. It is certainly important to be able to decide on the existence of tuberculosis when it *does* exist, and it is not less important to be able to decide correctly when it *does not* exist. A diagnosis early in the disease is also, for many reasons, highly desirable. The frequency with which cases wlll come under your observation, claiming decision upon the existence or non-existence of the disease, moreover, should commend the subject to your earnest study.

You are to arrive at a diagnosis by means of the combined testimony afforded by physical and rational methods of examination. Considered separately, physical, furnishes far more definite, reliable information than rational investigation; but as the object is to render our conclusion as certain and demonstrative as possible, neither method should be pursued to the exclusion of the other. We are to take a comprehensive view of all the facts, before forming an opinion; and in order that one may be qualified to do this, readily and satisfactorily, whenever cases present themselves, the various elements which the diagnosis involves are to be fully appreciated, singly and combined, and this knowledge is to be stored in the memory for practical application at any moment. It is an useful exercise here, as in other instances, occasionally to refresh the memory by recapitulating the several diagnostic points and considerations which the subject embraces. To assist you in so doing, with reference to the important disease selected as the subject for this occasion, has been my motive for submitting the foregoing hastily prepared remarks; and, by way of encouragement to the study of this subject, permit me to say, in conclusion, that, with due attention to the several signs and symptoms which I have enumerated and briefly commented on, you will experience but little difficulty in the diagnosis of pulmo-tuberculosis, even in a very early period of its existence, and under circumstances which will set at fault the judgment of those less familiar with the means of arriving at a correct conclusion. The satisfaction and advantage which you will derive from well grounded confidence in your ability to discriminate the disease, will sufficiently compensate for all the labor which proper qualification requires, without reference to other incentives of a higher and more disinterested character, which, I doubt not will both suggest and commend themselves to your reflections.

Feb. 1849.

